# Molecular Chaperone Hsp90 Is a Therapeutic Target for Noroviruses

**DOI:** 10.1128/JVI.00315-15

**Published:** 2015-04-08

**Authors:** Surender Vashist, Luis Urena, Mariam B. Gonzalez-Hernandez, Jayoung Choi, Alexis de Rougemont, Joana Rocha-Pereira, Johan Neyts, Seungmin Hwang, Christiane E. Wobus, Ian Goodfellow

**Affiliations:** a Division of Virology, Department of Pathology, University of Cambridge, Addenbrooke's Hospital, Cambridge, United Kingdom; b Section of Virology, Imperial College London, St. Mary's Campus, London, United Kingdom; c Department of Microbiology and Immunology, University of Michigan, Ann Arbor, Michigan, USA; d Department of Pathology, University of Chicago, Chicago, Illinois, USA; e National Reference Centre for Enteric Viruses, Public Hospital of Dijon, University of Bourgogne, Dijon, France; f KU Leuven–University of Leuven, Rega Institute for Medical Research, Leuven, Belgium

## Abstract

Human noroviruses (HuNoV) are a significant cause of acute gastroenteritis in the developed world, and yet our understanding of the molecular pathways involved in norovirus replication and pathogenesis has been limited by the inability to efficiently culture these viruses in the laboratory. Using the murine norovirus (MNV) model, we have recently identified a network of host factors that interact with the 5′ and 3′ extremities of the norovirus RNA genome. In addition to a number of well-known cellular RNA binding proteins, the molecular chaperone Hsp90 was identified as a component of the ribonucleoprotein complex. Here, we show that the inhibition of Hsp90 activity negatively impacts norovirus replication in cell culture. Small-molecule-mediated inhibition of Hsp90 activity using 17-DMAG (17-dimethylaminoethylamino-17-demethoxygeldanamycin) revealed that Hsp90 plays a pleiotropic role in the norovirus life cycle but that the stability of the viral capsid protein is integrally linked to Hsp90 activity. Furthermore, we demonstrate that both the MNV-1 and the HuNoV capsid proteins require Hsp90 activity for their stability and that targeting Hsp90 *in vivo* can significantly reduce virus replication. In summary, we demonstrate that targeting cellular proteostasis can inhibit norovirus replication, identifying a potential novel therapeutic target for the treatment of norovirus infections.

**IMPORTANCE** HuNoV are a major cause of acute gastroenteritis around the world. RNA viruses, including noroviruses, rely heavily on host cell proteins and pathways for all aspects of their life cycle. Here, we identify one such protein, the molecular chaperone Hsp90, as an important factor required during the norovirus life cycle. We demonstrate that both murine and human noroviruses require the activity of Hsp90 for the stability of their capsid proteins. Furthermore, we demonstrate that targeting Hsp90 activity *in vivo* using small molecule inhibitors also reduces infectious virus production. Given the considerable interest in the development of Hsp90 inhibitors for use in cancer therapeutics, we identify here a new target that could be explored for the development of antiviral strategies to control norovirus outbreaks and treat chronic norovirus infection in immunosuppressed patients.

## INTRODUCTION

Human noroviruses (HuNoV) are a major cause of viral epidemic gastroenteritis and a significant cause of morbidity and mortality worldwide (1–3). HuNoV are mainly transmitted through the fecal-oral route (4). Although norovirus disease is usually short-lived and self-limiting, immunocompromised patients can become chronically infected, with symptoms lasting for weeks or years (5, 6). Despite significant efforts, HuNoV have yet to be cultured efficiently in the laboratory. However, our understanding of their biology has improved significantly in recent years (7, 8), and a recent study has demonstrated limited replication in immortalized B cells in the presence of enteric bacteria (9). Some of these advances have been due to the discovery of a murine norovirus (MNV), which can be cultivated in cell culture (10). Reverse-genetics approaches and small animal models for MNV have enabled better insights into the norovirus life cycle, including the identification of cellular factors and pathways involved in norovirus replication (11–14).

As obligate intracellular parasites, viruses rely heavily on the cellular pathways and processes for almost every step of their life cycle. RNA viruses typically have small genomes and therefore possess limited coding capacity. As a result, RNA viruses typically utilize numerous mechanisms to increase the information contained within their genomes. These mechanisms include the use of translational frameshifting and other novel translation mechanisms, as well as the incorporation of large polyproteins, which are processed to produce precursors, as well as the final mature protein products, each of which can have different functional properties (15, 16). In addition, the genomes of RNA viruses invariably fold to form RNA structures that interact with both cellular and viral proteins to regulate many aspects of the viral life cycle. These types of interactions are known to contribute to genome replication, translation, encapsidation, and pathogenesis (17–23). For example, a single nucleotide change in the poliovirus internal ribosome entry site (IRES) structure leads to the attenuation of virus in a cell-specific manner (24, 25), whereas the deletion of a polypyrimidine tract in the MNV 3′-untranslated region (3′UTR) leads to attenuation of virus *in vivo* (26). The identification and inhibition of these viral RNA-host protein interactions have been shown to have the potential to control viral infections (27, 28). Targeting a host protein has the additional advantage of a high genetic barrier to drug resistance due to the extremely low mutational rate of eukaryotic cells. In contrast, RNA viruses encode RNA-dependent RNA polymerases which lack proofreading capacity, leading to the generation of a mutant spectra referred to as “quasispecies,” greatly facilitating the emergence of drug resistance (29–31).

Using a riboproteomics-based approach, we recently identified heat shock protein 90 (Hsp90) as one component of a network of host proteins that interact directly or indirectly with the 5′ and 3′ extremities of the MNV-1 genome (32). Given the multifunctional nature of viral proteins, it is not surprising that Hsp90 appears to play a role in the life cycles of many pathogenic viruses and that targeting Hsp90 can effectively control virus replication in cell culture (33). In the present study, we have further investigated the role of Hsp90 in the norovirus life cycle and, in addition to our observed RNA binding activity of Hsp90, we identified that the major capsid protein VP1 of both MNV-1 and HuNoV are Hsp90 client proteins. Importantly, we further demonstrate that inhibiting Hsp90 negatively impacts norovirus replication both in cell culture and *in vivo*. We provide here a proof of principle that targeting a molecular chaperone may provide a therapeutic approach for treating or controlling norovirus infections.

## MATERIALS AND METHODS

### Cell culture and preparation of BMDMs.

The murine microglial BV-2 and 293T cell lines were maintained in Dulbecco modified Eagle medium (Gibco) supplemented with 10% fetal calf serum (Biosera), 2 mM l-glutamine (Gibco), and antibiotics (100 U of penicillin/ml and 100 μg of streptomycin/ml). Baby hamster kidney cells expressing T7 DNA polymerase (BSRT7 cells) were cultured in medium described above containing G418 at a concentration of 1 mg/ml. Primary bone marrow-derived macrophages (BMDMs) were prepared from *Atg5*^flox/flox^ and *Atg5*^flox/flox+*LysMcre*^ mice as described previously (34).

### Virus and reagents.

The MNV-1 CW1 strain was used for all cell culture experiments, whereas the CW3 strain was used for the infection of mice. Neutral red (NR)-containing viral stocks were prepared in the absence of white light as described previously (35, 36). Antisera against viral proteins were developed in the laboratory and were described previously (32, 37). Antibodies against host factors were purchased from the following suppliers: Akt (Santa Cruz, sc-8312), Hsp90 (Santa Cruz, sc-59578), LC3B (Sigma, L7543), and beta-actin (Sigma, A5316).The inhibitors 17-dimethylaminoethylamino-17-demethoxygeldanamycin (17-DMAG) and 17-allylamino-17-demethoxygeldanamycin (17-AAG) were purchased from Selleckbio, lactacystin was purchased from Cayman Chemicals, and novobiocin was purchased from Cambridge Biosciences. The chemicals were used at the concentrations indicated in the text. His-tagged recombinant Hsp90 was purchased from Cayman Chemicals, whereas recombinant His-tagged glutathione *S*-transferase (GST) protein was provided by Stephen Graham, University of Cambridge. His-tagged recombinant polypyrimidine-binding protein (PTB) and viral protein NS7 were prepared as described previously (38, 39). The plasmids pT7MNV:Rz, pSG, and pMNV-1 VP1 expressing MNV-1 genomic RNA, subgenomic RNA, and VP1 genes, respectively, as well as the plasmid pHuNOV VP1 expressing HuNoV VP1 gene, were cloned under T7 promoter in either pTriex or pcDNA3.1 vectors. BSRT7 cells were infected with fowlpox virus expressing T7 RNA polymerase before transfections as described earlier (13). A plasmid expressing green fluorescent protein (GFP)-Hsp90α was a gift from Edouard Bertrand and was described previously (40). Where noted, cells were transfected using Lipofectamine 2000 according to the manufacturer's instructions (Life Technology).

### Enrichment of RNA binding proteins using RNA affinity purification.

RNA affinity purifications were performed as described previously (32). Briefly, RNA transcripts representing 5′ and 3′ extremities of MNV-1 genome and a control RNA representing a portion of the ampicillin resistance gene were prepared by *in vitro* transcription and covalently coupled to cyanogen bromide-activated Sepharose. The RNA-linked Sepharose were then incubated with 7.5 mg of BV-2 S-100 lysates in the presence of 100 μg of yeast RNA, 1 mM ATP, 1 mM GTP, and 100 U of RNasin RNase inhibitor (Promega) at 4°C for 2 h with continuous mixing. RNA-protein complexes were washed three times with binding buffer at 4°C, and proteins were eluted by boiling in the presence reducing SDS-PAGE loading buffer. The eluted proteins were analyzed by Western blot analysis.

### Recombinant Hsp90 interaction with viral RNA.

Ni-NTA resin (20 μl of packed volume), pre-equilibrated with NT2 buffer (50 mM Tris [pH 7.4], 150 mM NaCl, 1 mM MgCl_2_, 0.05% Nonidet P-40), was incubated with various His-tagged proteins in the presence of protease inhibitor at 4°C. After 2 h, resin was washed twice with ice-cold NT2 buffer and incubated with 10 μg of total RNA isolated from MNV-1-infected cells in the presence of 100 U of RNaseOUT and 10 μg of yeast RNA at 4°C. After 2 h, the complexes were washed twice with ice-cold NT2 buffer, followed by RNA extraction using a GenElute mammalian total RNA miniprep kit (Sigma). RNA samples were analyzed by reverse transcription-quantitative PCR (RT-qPCR) analysis according to a previously published protocol (41). For semiquantitative analysis, RT-PCR was performed as described previously (32). Briefly, a 1/10 portion of the RNA was reverse transcribed to cDNA using primer IGIC464 (CAAACATCTTTCCCTTGTTC) targeting nucleotides (nt) 1779 to 1760 of the MNV-1 genome utilizing M-MLV reverse transcriptase (Promega) according to the manufacturer's protocol. Then, 1/10 of the cDNA was subjected to PCR with the primers IGIC200 (AACGCTCTGCTGGCCAGGATCAGC) targeting nt 1416 to 1439 and primer IGIC464 to produce a 364-bp product encompassing nt 1416 to 1779 of the MNV-1 genome.

### Detection of viral RNA using qRT-PCR.

RNA was reverse transcribed to cDNA by using oligonucleotide IC464 (CAAACATCTTTCCCTTGTTC) and AMV reverse transcriptase (Promega) as detailed in the manufacturer's instructions. cDNA was amplified by real-time PCR using qPCR master mix (Eurogentec) on an ABI 7900 real-time PCR machine. Briefly, cDNA was mixed with 2× buffer and the primers IC464 and IC465 (TGGACAACGTGGTGAAGGAT), heat denatured to 95°C for 10 min, and subjected to 40 cycles of 94°C for 15 s, 58°C for 30 s, and 72°C for 30 s. The viral genome copy number was calculated by interpolation from a standard curve generated using serial dilutions of standard RNA representing nt 1085 to 1986 of the MNV-1 genome.

### Virus titer determination.

Virus titers were quantified by plaque assay or a 50% tissue culture infective dose (TCID_50_) assay as described earlier (42, 43).

### IC_50_ and CC_50_ determination.

The cytotoxicity of 17-DMAG for BV-2 cells was determined by using a CellTiter-Blue cell viability assay (Promega) according to the manufacturer's guidelines. Briefly, BV-2 cells were plated in quadruplicate at a density of 10^4^ cells per well in a 96-well plate in the presence of various concentrations of 17-DMAG. After 24 h of incubation, 20 μl of CellTiter-Blue reagent was added to the wells, the incubation was continued for another 4 h, and the absorbance was measured at 570 and 600 nm. The final absorbance (*A*_570–600_) was calculated by subtracting average culture medium absorbance at 600 nm from the total absorbance values of experimental wells at 570 nm. A dose-response curve was plotted using the *A*_570–600_ values of untreated cells as 100%. The 50% cytotoxic concentration (CC_50_) was calculated from the dose-response curve using nonlinear regression analysis. 17-DMAG displayed an CC_50_ of 4.5 μM. In order to determine the potency of the inhibitory effect of 17-DMAG on MNV-1, the 50% inhibitory concentration (IC_50_) in BV-2 cells was determined after 24 h of treatment. Briefly, the BV-2 cells were infected with MNV-1 (multiplicity of infection [MOI], 5 TCID_50_/cell) and incubated with various concentration of drugs 17-DMAG for 24 h. Samples were harvested for virus titer determinations using TCID_50_. The dose-response curve was plotted using the virus titer of untreated cells as 100%, and the IC_50_ was calculated using nonlinear regression analysis. 17-DMAG displayed an IC_50_ of 0.06 μM during single cycle MNV-1 replication in BV-2 cells. The selectivity index (SI; SI = CC_50_/IC_50_) was calculated to be 69.75.

### Lumier assay.

Murine and human Hsp90α genes were cloned by RT-PCR from the total mRNAs isolated from the BV2 and HEK293T cell lines, respectively. All MNV and HuNoV genes encoding individual proteins were cloned from MNV-1 and Norwalk strains, respectively. N-terminal Renilla *reniformis* luciferase (RL) or S. aureus protein A (PA) fusion protein plasmids were generated in pcDNA3-RL-GW and pTrex-Dest30-PA expression vectors, respectively (a gift from Juergen Haas, University of Edinburgh). Lumier assays were performed in quadruplicate as previously described using sheep anti-rabbit IgG-coated magnetic beads (Invitrogen, Dynabeads M280) and a modified HENG lysis buffer [20 mM HEPES (pH 7.8), 135 mM KCl, 2 mM MgCl_2_, 15 mM (NH_4_)_6_Mo_7_O_24_, 0.5% NP-40, 0.1% bovine serum albumin, 1 mM ATP, 1 mM dithiothreitol] with 1% Halt protease/phosphatase inhibitor cocktail (Thermo Scientific) and benzonase (Novagen) (44). Negative and positive thresholds were determined by using a plasmid expressing the luciferase fusion protein cotransfected with a plasmid construct expressing PA alone. After 24 h of incubation, the cells were lysed on a shaker using 30 μl of ice-cold lysis buffer for 10 min at 4°C. Protein expression was confirmed in HEK293T cells by Western blotting. Lysates were centrifuged for 15 min at 1,000 × g at 4°C and transferred to a 96-well white flat-bottom plate (Greiner) with beads, incubated for 1 h on a shaker at 4°C, and then washed six times on a handheld magnetic separator block from Millipore. After washing, the Renilla activity was measured on a GloMax luminometer (Promega). Normalized Lumier intensity ratios (NLIR) and robust z (rz) scores were calculated as previously described (45). An rz score above 3.72 (*P* > 0.0001) was considered positive.

### RNA interference reduction of Hsp90 levels.

MNV-1 permissive BV-2 cells were used for small interfering RNA (siRNA)-mediated protein knockdown as described previously (32). Briefly, 6 × 10^6^ BV-2 cells were electroporated with 100 pmol of a pool of siRNAs against Hsp90α/β (sc-35610; Santa Cruz) at 1,700 V for 10 ms for three pulses using the Neon transfection system (Invitrogen). The cells were plated in antibiotic-free medium to allow recovery and incubated for 12 h. The cells were then infected with MNV-1 (MOI, 0.01 TCID_50_/cell), and samples were harvested for RNA isolation, virus titer determinations, and Western blotting at various time points postinfection. The virus titer was determined by determining the TCID_50_, and the viral RNA copy number was quantified using RT-qPCR as described previously (32).

### *In vivo* inhibition of MNV-1.

All of the animal studies described here were performed in accordance with local and federal guidelines as outlined in the *Guide for the Care and Use of Laboratory Animals* of the National Institutes of Health. Protocols were approved by the University of Michigan Committee on Use and Care of Animals (UCUCA PRO00004534). 6- to 8-week-old BALB/c mice were purchased from Jackson Laboratories and housed at the University of Michigan. Mice were administered 30 mg/kg of 17-DMAG dissolved in 0.05% dimethyl sulfoxide (DMSO) or vehicle control intraperitoneally 1 h prior to infection with light-sensitive, neutral red (NR) MNV-1 (10^6^ PFU) by oral gavage. The distal ileum was harvested in the dark after 24 h, and the virus titer was determined by plaque assay as previously described (35, 36).

## RESULTS

### Hsp90 interacts with the termini of the MNV-1 genome.

**FIG 1:**
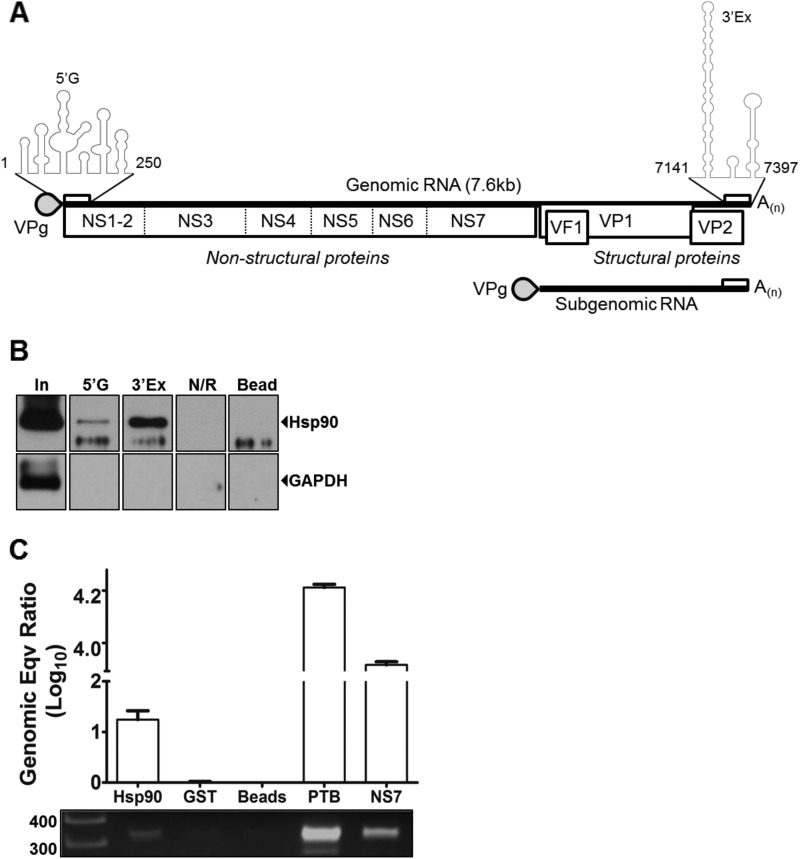
Hsp90 interacts with the MNV-1 genomic RNA. (A) Murine norovirus genome organization and the encoded proteins are presented. The secondary structure of 5′ (5′G) and 3′ extremities (3′Ex) with which Hsp90 interacts has been presented as a folded structure. (B) Hsp90 is enriched on the RNA affinity columns representing the 5′ and 3′ extremities of the MNV-1 genome. RNA affinity columns representing 5′G, 3′Ex, and a control RNA (N/R), as well as beads alone, were used to purify the host factors from BV-2 cells. The enriched proteins were examined by Western blot analysis to detect enrichment of Hsp90 and GAPDH (glyceraldehyde-3-phosphate dehydrogenase). (C) Recombinant Hsp90 interacts specifically with the MNV-1 genome. His-tagged recombinant proteins (Hsp90, GST, PTB, and NS7) coupled to Ni-NTA beads were tested for its ability to bind positive-strand MNV-1 genome. The bound viral RNA enriched on these proteins was quantified by using RT-qPCR and expressed relative to bead controls. Bound viral RNA was also amplified by using endpoint RT-PCR and detected on an agarose gel. The experiment was performed in triplicate, and the error bars represent standard deviations.

The termini of MNV genomes are highly conserved between strains and fold into characteristic structures that we have shown to be required for viral replication (Fig. 1A). We have previously identified Hsp90α/β as a host factor that interacts with the 5′ and 3′ termini of the MNV-1 genome using RNA affinity chromatography and mass spectrometry (32). Many of the host proteins also identified within the network of interacting proteins are known Hsp90 client proteins (33). To independently corroborate the binding of Hsp90 with the MNV-1 genome, we performed RNA affinity chromatography as previously described (32), and the presence of Hsp90 was verified by Western blot analysis of the eluted fractions. Hsp90 was enriched following RNA affinity purification on the 5′ as well as the 3′ extremity of the MNV-1 genomic RNA (Fig. 1B). Hsp90 was not enriched on a control RNA (N/R) or with beads alone. To determine whether Hsp90 associated with viral RNA, we examined the ability of recombinant His-tagged Hsp90 (rHsp90) to enrich MNV-1 genomic RNA from total cellular RNA isolated from MNV-1-infected cells. Positive-sense viral RNA was selectively enriched by immobilized rHsp90, as well as with His-tagged poly-PTB and the viral polymerase NS7 (Fig. 1C). In contrast, negative-sense viral RNA was not enriched by rHsp90 (data not shown). This observation suggests an interaction of Hsp90 with viral RNA and agrees with previous observations describing viral RNA binding activity of Hsp90 in bamboo mosaic virus (46).

### MNV-1 growth is dependent on Hsp90 levels in cell culture system.

**FIG 2:**
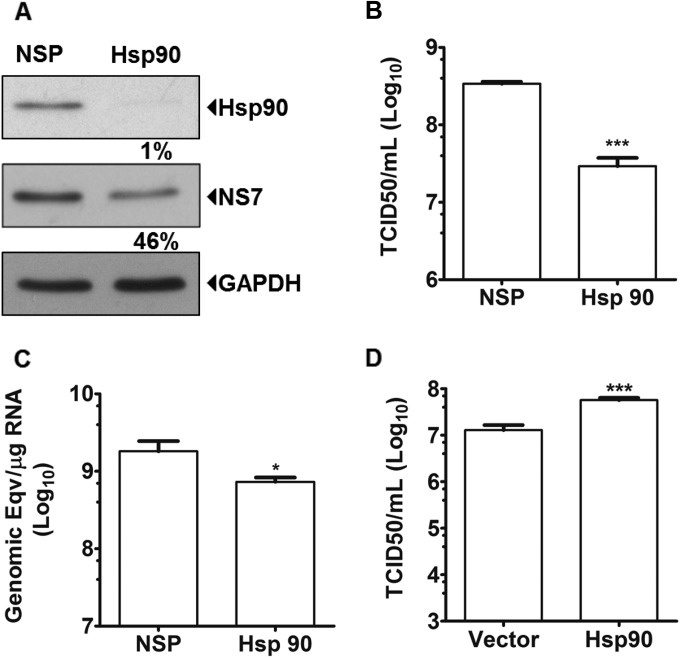
Hsp90 activity is required for MNV-1 replication. BV-2 cells were transfected with either nonspecific (NSP) siRNA duplexes or siRNA targeting Hsp90 and infected with MNV-1 at an MOI of 0.05 TCID_50_ per cell. Samples were harvested at 24 h postinfection for analysis. (A) The levels of protein in each sample were analyzed by Western blot and quantified by using densitometry. Samples were normalized to the level of GAPDH. Hsp90 and NS7 protein levels in Hsp90 siRNA transfected cells are expressed relative to the levels in NSP siRNA transfected cells. (B) Total infectious virus production was determined by TCID50. (C) Levels of positive sense viral RNA determined were determined by RT qPCR. (D) VPg linked MNV-1 RNA was transfected in 293T cells overexpressing GFP-Hsp90 and the virus titer was determined 24 h later using the TCID_50_. Each experiment was performed in triplicate, and the error bars represent standard deviations. Statistical analysis was performed using by one-way analysis of variance (ANOVA) with Bonferroni's post test (*, *P* < 0.05; ***, *P* < 0.001).

To determine whether Hsp90 was required for some aspect of the norovirus life cycle, we examined the effects of siRNA-mediated reduction of intracellular Hsp90α/β levels on MNV-1 replication. siRNA-mediated targeting of Hsp90 resulted in a >99% reduction in Hsp90 levels, although detectable levels of Hsp90 remained (Fig. 2A). Cells transfected with Hsp90 siRNA were then infected with MNV-1 and the levels of viral RNA, viral proteins, and infectious virus produced were examined using RT-qPCR, Western blot, and TCID_50_ assays, respectively. Viral RNA polymerase (NS7) production was reduced by >50% (Fig. 2A), whereas genomic RNA levels, as well as virus titers, were reduced significantly (Fig. 2B and C). In addition, overexpression of Hsp90α in 293T cells resulted in a statistically significant increase (>4-fold) in MNV-1 titers after transfection of purified VPg-linked MNV-1 RNA (Fig. 2D). Taken together, these data confirmed a requirement for Hsp90 in some aspect of the MNV-1 life cycle.

### Hsp90 activity is required for MNV-1 replication.

**FIG 3:**
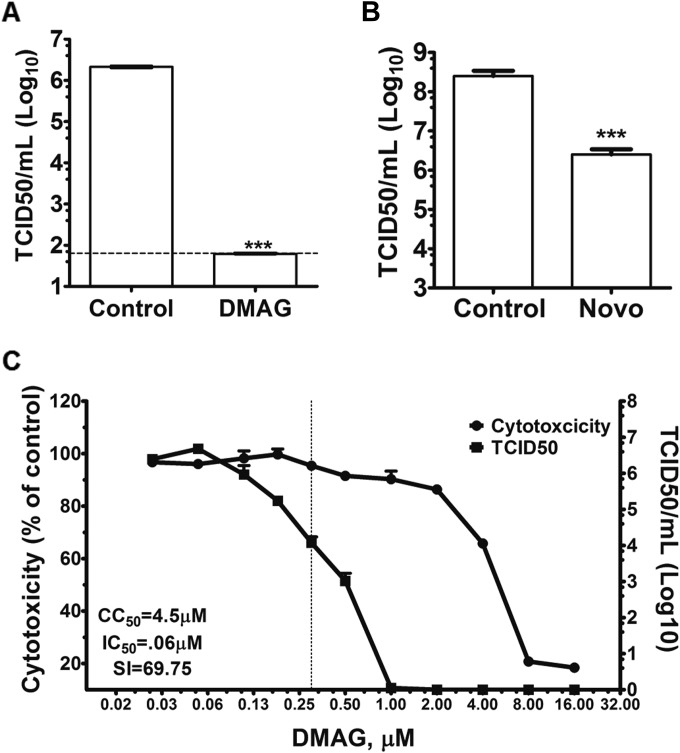
Hsp90 activity is required for MNV-1 replication. The requirement of Hsp90 activity for MNV-1 replication was assessed by using Hsp90 activity inhibitor and overexpression. (A) BV-2 cells were infected with MNV-1 at a low MOI (0.01 TCID_50_/cell) and treated with 17-DMAG. Samples were harvested 24 h postinfection, and virus titers were determined by a TCID_50_ assay. (B) Novobiocin, a small molecule that interacts with C-terminal ATP binding site of Hsp90 to inhibit its activity, was used to confirm Hsp90-mediated inhibition of MNV-1. BV-2 cells were infected with MNV-1, and the medium was supplemented with novobiocin. At 12 h postinfection, samples were harvested to determine virus titers by a TCID_50_ assay. (C) The cytotoxicity of 17-DMAG on BV-2 cells was determined by using a CellTiter-Blue assay, and the inhibitory effect of 17-DMAG on MNV-1 replication was determined using the TCID_50_ during a high-MOI (5.0 TCID_50_/cell) infection. A concentration-effect curve was plotted. The 50% cytotoxic concentration (CC_50_) and the 50% inhibitory concentration (IC_50_) were calculated from the curve after nonlinear regression analysis. The concentration of 17-DMAG used during further studies is indicated as a dotted line. Each experiment was performed in triplicate, and the error bars represent standard deviations. Statistical analysis was performed using two-way ANOVA with Bonferroni's post test (***, *P* < 0.001).

Small molecule inhibitors have been widely used to study the function of Hsp90, as well as its role in the life cycle of several viruses (17, 33, 47–49). We examined the effect of the water-soluble Hsp90 inhibitor 17-DMAG on MNV-1 replication in cell culture (Fig. 3). 17-DMAG is a geldanamycin (GA) analogue that binds to the ATP-binding site in the N-terminal region of Hsp90, inhibiting chaperone activity. We examined the ability of 17-DMAG to interfere with MNV-1 replication in a virus yield assay, wherein cells were infected at a low MOI (0.01 TCID_50_/cell), and the effect on infectious virus yield at 24 h postinfection was determined. Treatment of cells with 0.3 μM 17-DMAG reduced the levels of infectious MNV-1 produced by >4 orders of magnitude without significant cytotoxicity (Fig. 3A). Treatment with 17-AAG, another analogue of GA, was also found to significantly reduce infectious virus production using the same approach (data not shown).

To further confirm that the effect observed on MNV-1 replication is due to a direct inhibition of Hsp90 activity by 17-DMAG, we also examined the effect of another unrelated inhibitor, novobiocin, on MNV-1 replication. Novobiocin is an aminocoumarin antibiotic, exhibiting potent activity against Gram-positive bacteria and therefore structurally unrelated to 17-DMAG. In contrast to 17-DMAG, which binds the N terminus of Hsp90, novobiocin interacts with the C-terminal ATP binding site (50–52). Novobiocin treatment of cells also resulted in a significant decrease in MNV-1 titers (Fig. 3B) without any overt signs of toxicity (data not shown). These combined results suggested that the inhibition of Hsp90 activity significantly reduces MNV-1 replication in cell culture.

Due to high water solubility and favorable pharmacokinetic properties, all further assays were performed with 17-DMAG. A concentration of 0.3 μM and a high MOI (5.0 TCID_50_/cell) was used for all further experiments based on the concentration-effect curve (Fig. 3C), since this concentration caused no toxicity while being highly effective (>2.5 log_10_) for the inhibition of MNV-1 replication.

### Hsp90 activity is required for norovirus replication at a postentry step.

**FIG 4:**
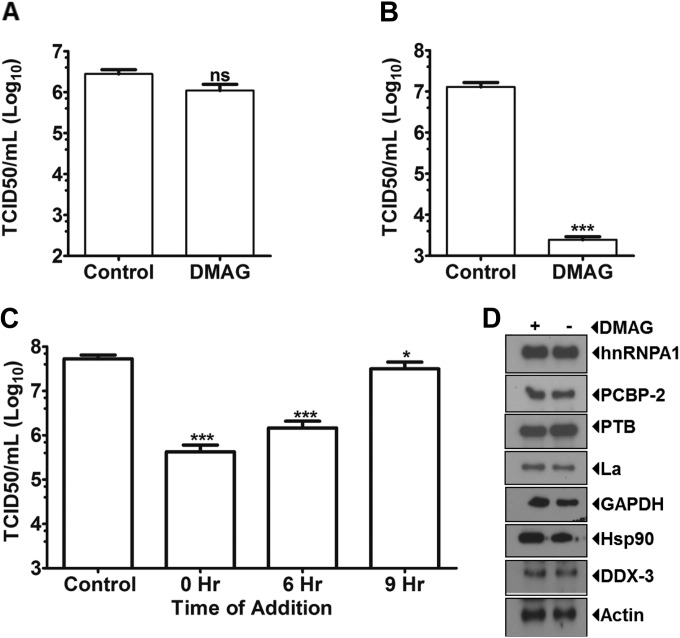
17-DMAG affects MNV-1 replication after viral entry. 17-DMAG was tested for its ability to inhibit MNV-1 infection at various time points postinfection. (A) To confirm that treatment with 17-DMAG does not affect MNV-1 virion stability and its infectivity, MNV-1 stock was treated for 1 h with 17-DMAG, diluted, and titrated by using a TCID_50_ assay. (B) VPg-linked MNV-1 RNA isolated from infected cells was transfected in 293T cells. After 4 h, the cells were washed and supplemented with 17-DMAG or phosphate-buffered saline-containing media. Samples were harvested after 24 h and virus titers were plotted as determined by the TCID_50_. (C) BV-2 cells were infected with MNV-1 at an MOI of 5 TCID_50_/cell, and 17-DMAG was added at various times postinfection. 17-DMAG significantly inhibits MNV-1 replication up to 9 h postinfection. (D) The effect of 17-DMAG on the levels of host proteins known to play a role in MNV-1 infection was determined by using Western blotting at 12 h postinfection. Each experiment was performed in triplicate, and the error bars represent standard deviations. Statistical analysis was performed by two-way ANOVA with Bonferroni's post test (ns, nonsignificant; *, *P* < 0.05; ***, *P* < 0.001).

To rule out the possibility that 17-DMAG treatment resulted in direct inactivation of MNV-1 virions, the effect of the inhibitor directly on virus viability was assayed. MNV-1 virus stocks were incubated with 17-DMAG prior to extensive dilution of the samples for titration. Pretreatment of MNV-1 with 17-DMAG had no effect on the titers of the treated stocks, indicating that 17-DMAG did not inactivate virions directly (Fig. 4A). To examine a role for Hsp90 in MNV-1 entry or uncoating, the effect of 17-DMAG on virus replication after transfection of VPg-linked viral RNA into HEK293T cells was examined. Importantly, since human cells are not susceptible to MNV-1 infection due to lack of a suitable receptor (13), infectious titers from human cells represent a single cycle only. Inhibition of Hsp90 activity in HEK293T cells by 17-DMAG resulted in a >3-log_10_ reduction in virus titer (Fig. 4B). A similar reduction in titer was observed during MNV-1 recovery from an infectious cDNA clone in BHK cells in the presence of 17-DMAG (data not shown). These data indicated that Hsp90 activity functions at a postentry stage of the MNV-1 life cycle.

In order to further delineate the role of Hsp90 in the MNV-1 life cycle, we examined the effect of 17-DMAG addition at various times postinfection (0, 6, and 9 h). Virus infections were performed using a high MOI to ensure a single cycle of synchronous replication. We observed that inhibition of Hsp90 activity had maximal effect on MNV-1 titer when 17-DMAG was added either at 0 or 6 h postinfection. However, a small, yet statistically significant reduction in infectious virus production could be observed even when 17-DMAG was added at 9 h postinfection (Fig. 4C). These data again suggested that Hsp90 activity was required at a postentry stage of the MNV-1 life cycle. To control for any nonspecific effects of Hsp90 inhibition on the MNV-1 replication, we examined the levels of cellular proteins that have previously been identified as being involved in the norovirus life cycle (Fig. 4D). Inhibition of Hsp90 activity did not affect the levels of any of the cellular proteins examined.

### Inhibition of Hsp90 activity leads to the inability of MNV-1 genome to be encapsidated.

**FIG 5:**
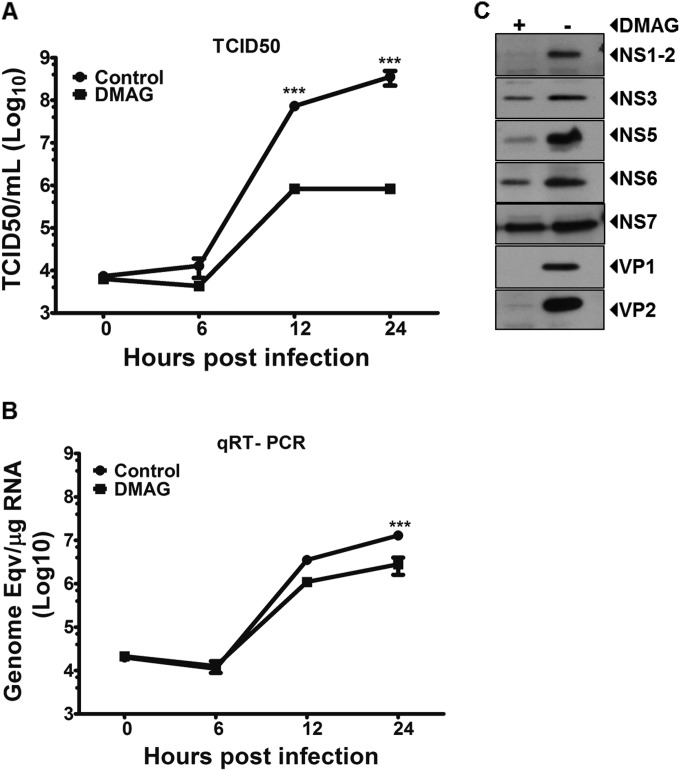
Inhibition of Hsp90 activity leads to the inability of MNV-1 genome to be encapsidated. A one-step growth curve of MNV-1 was carried out to analyze the effect of 17-DMAG on virus titer (A) and genome copy number (B). (C) The effect of 17-DMAG on the levels of viral proteins was determined by using Western blotting at 12 h postinfection. Each experiment was performed in triplicate, and the error bars represent the standard deviations. Statistical analysis was performed using by two-way ANOVA with Bonferroni's post test (***, *P* < 0.001).

To define the precise role of Hsp90 in the norovirus life cycle, the effect of 17-DMAG was examined using a high-multiplicity single-step growth curve. As expected from our previous observations, Hsp90 activity inhibition using 0.3 μM 17-DMAG resulted in decreased virus titer by up to ∼3 log_10_ (Fig. 5A). In contrast, viral RNA levels were decreased only at the later stages of infection and by up to ∼1 log_10_ only (Fig. 5B). These observations suggested that although Hsp90 activity contributes to viral RNA replication, the primary function of Hsp90 appears to be at a late stage of the viral life cycle, after viral RNA synthesis has occurred. Hsp90 is a molecular chaperone, and its activity is known to be required for the correct folding of a diverse number of viral proteins. Inhibition of Hsp90 activity typically leads to the specific degradation of these viral proteins (33, 53, 54). We therefore examined the effect of 17-DMAG on the levels of MNV-1 structural and nonstructural proteins during infection (Fig. 5C) under the same conditions where cellular protein levels were unaffected (see Fig. 4D). The structural proteins VP1 and VP2 were almost undetectable in the presence of 17-DMAG at 12 h postinfection. As expected, due to the decrease in viral RNA levels, a minor decrease in nonstructural protein levels was also observed, although it is worth noting that not all proteins appeared to be equally affected; for example, the levels of NS6 and NS7 were less affected than NS1-2. These data, combined with the observation that inhibition of Hsp90 activity has a greater impact on viral infectivity than any observed effect on RNA synthesis, suggested that Hsp90 activity contributes directly to the formation of an infectious viral particle by regulating the stability of the structural proteins.

### The major capsid protein VP1 is a client protein of Hsp90.

**FIG 6:**
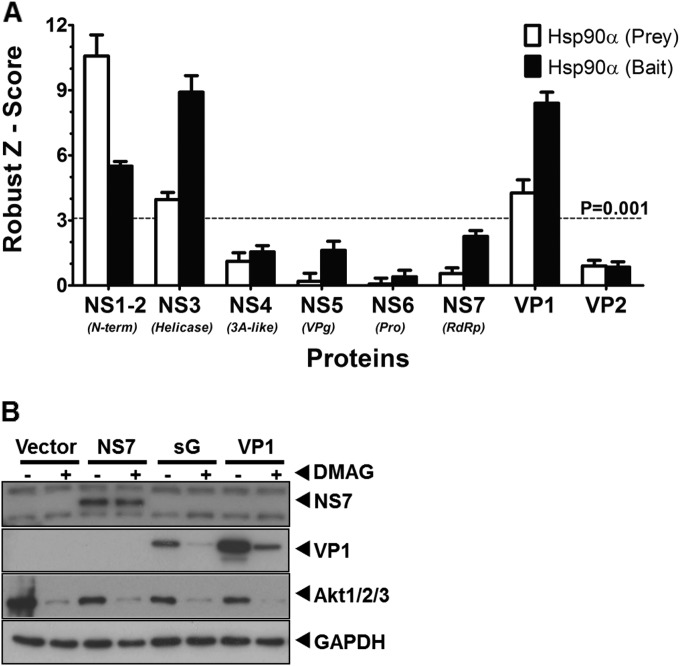
Hsp90 interacts with MNV-1 capsid protein and inhibition of Hsp90 activity leads to its degradation. (A) Lumier assay was used to detect direct interaction of Hsp90 with MNV-1 proteins in 293T cells. MNV-1 proteins and murine hsp90α were expressed in fusion with either protein A (bait) or Renilla luciferase (prey). The expressed proteins were pulled down using IgG, and the interacting fusion Renilla luciferase activity was measured. The assay was performed using Hsp90 as both prey and bait. The robust z scores were calculated to measure the significance of interaction. The experiment was performed in triplicate, and the error bars represent the standard deviations. (B) MNV-1 nonstructural (NS7) and structural (VP1) proteins were expressed in BSR-T7 cells and tested for the ability to be degraded in the presence of 17-DMAG. An Hsp90 client protein, Akt, and GAPDH were used as positive and loading controls, respectively. NS7 levels remained similar, whereas VP1 levels were reduced in the presence of 17-DMAG.

Given the observation that inhibition of Hsp90 activity resulted in the almost complete loss of several viral proteins, we sought to determine whether any of the viral proteins were Hsp90 client proteins and interacted directly with Hsp90. The Lumier quantitative immunoprecipitation assay (55) was used to examine potential interaction between Hsp90α and the various proteins encoded by MNV-1. Lumier was selected due to the ability to detect low-affinity, transient interactions, often seen between Hsp90 and client proteins. Bait protein were fused to protein A and prey proteins fused to Renilla luciferase. After expression in cells, bait proteins were purified from cells using IgG bound to magnetic beads, along with any associated luciferase-tagged prey proteins. The normalized Lumier intensity ratios (NLIR) and rz score for these NLIR values for each interaction pair was calculated as described in Materials and Methods. The rz scores for each interaction, a measure of the statistical significance of each interaction (56), confirmed that interaction of Hsp90α with NS1-2, NS3, and VP1 was statistically significant (rz score > 3.72 = *P* < 0.0001) (Fig. 6A).

Based on the combination of data obtained to this point, we reasoned that the antiviral activity of Hsp90 inhibitors was primarily mediated by an effect on the viral capsid protein VP1. Therefore, to further confirm VP1 as a Hsp90 client protein, the effect of inhibiting Hsp90 activity on the stability of VP1 was then examined outside the context of viral infection. The MNV-1 structural protein VP1 was overexpressed in cells either alone or with VP2 from the MNV-1 subgenomic RNA. Samples treated with 17-DMAG showed a reduced level of VP1, whether expressed alone or with VP2 (Fig. 6B), whereas the level of NS7 remained unaltered. Akt-1 is a well-known client protein of Hsp90 and was used as positive control to confirm the effective inhibition of Hsp90 activity (57). Taken together, these data independently corroborated that the MNV-1 structural protein VP1 interacts with Hsp90 and that its levels are reduced by inhibition of Hsp90 activity, confirming that VP1 is an Hsp90 client protein.

### After inhibition of Hsp90, the MNV-1 VP1 protein is not degraded by a proteasomal or autophagy pathway.

**FIG 7:**
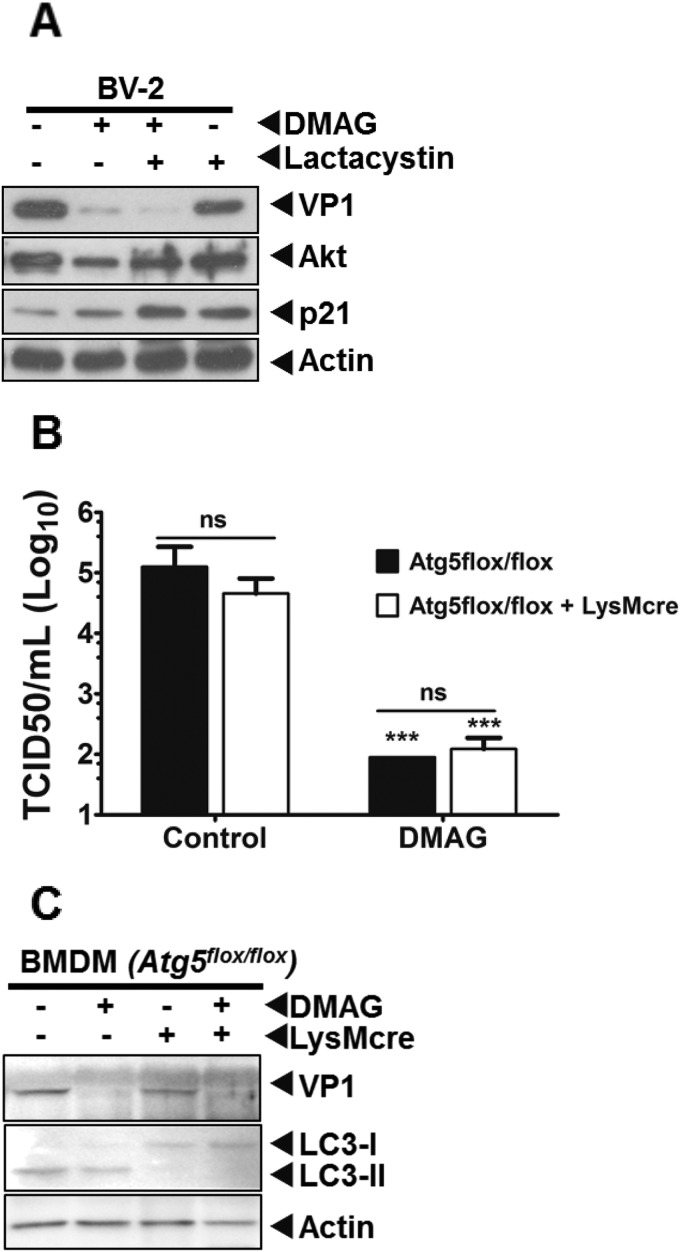
Autophagy or proteasome activity is not involved in VP1 degradation. (A) The effect of specific inhibitor of proteasome activity, lactacystin, on the downregulation of MNV-1 protein levels due to 17-DMAG was examined using Western blot analysis. P21, a cellular protein degraded by the proteasome, was used as a positive control to confirm lactacystin activity in BV-2 cells. Akt-1, an Hsp90 client protein that is degraded by proteasome activity, was used as a positive control to confirm the rescue the effect by inhibition of proteasome activity. (B) Atg5-deficient (*Atg5*^flox/flox^+*LysMcre*) BMDMs were used to examine the role of autophagy in the degradation of MNV-1 capsid protein. Atg5-deficient and control (*Atg5*^flox/flox^) BMDMs were infected with MNV-1 at an MOI of 5 and treated with 17-DMAG. Samples were harvested at 24 h postinfection for virus titer and protein level determinations. The virus titer was determined by using the TCID_50_. The experiment was performed in triplicate, and the error bars represent the standard deviations. (C) The levels of MNV-1 capsid protein VP1 and the autophagy markers, LC3-I and LC3-II, were determined by Western blot analysis. The conversion of LC3-I to LC3-II is blocked in Atg5-deficient BMDMs, but the levels of VP1 are not rescued.

When Hsp90 activity is inhibited, client proteins are typically degraded via one of the several host cell quality control mechanisms (58–60). Inhibition of the degradative pathway involved in the loss of VP1 after Hsp90 inhibition should therefore result in the restoration of the VP1 protein levels. We thus examined the effect of inhibition of the major cellular degradation pathways, namely, the proteasome or autophagic pathways, in order to determine whether either of these processes played a role in degradation of the MNV-1 VP1 protein upon Hsp90 inhibition. Lactacystin, a well-characterized, specific proteasome inhibitor, was used to examine the role of the proteasome in the degradation of MNV-1 structural protein VP1 after inhibition of Hsp90 activity. Treatment with lactacystin failed to rescue 17-DMAG mediated reduction in the levels of VP1 protein in MNV-1-infected BV-2 cells (Fig. 7A). The accumulation of p21, a cellular protein continuously degraded by proteasome, was used as indicator of proteasome activity inhibition. We also investigated whether autophagy was involved in 17-DMAG-mediated reduction in the levels of VP1 using Atg5 knockout BMDMs. MNV-1 replicated to similar titers in both Atg5-deficient (*Atg5*^flox/flox+*LysMcre*^) and wild-type (*Atg5*^flox/flox^) BMDMs (34). Inhibition of Hsp90 activity using 17-DMAG reduced virus titers significantly in both Atg5-deficient and wild-type BMDMs (Fig. 7B). Similarly, the levels of viral protein VP1 were not rescued in Atg5-deficient BMDMs (Fig. 7C). These results indicated that Hsp90 inhibition-mediated degradation of MNV-1 protein VP1 is not mediated via either proteasome or autophagy. Furthermore, VP1 protein translation occurred in the presence of 17-DMAG (data not shown), suggesting MNV-1 VP1 is most likely degraded by a nonproteasomal and nonautophagy pathway. However, identification of the degradation pathway for VP1 requires further investigations.

### Hsp90 activity is required for the MNV-1 replication *in vivo*.

**FIG 8:**
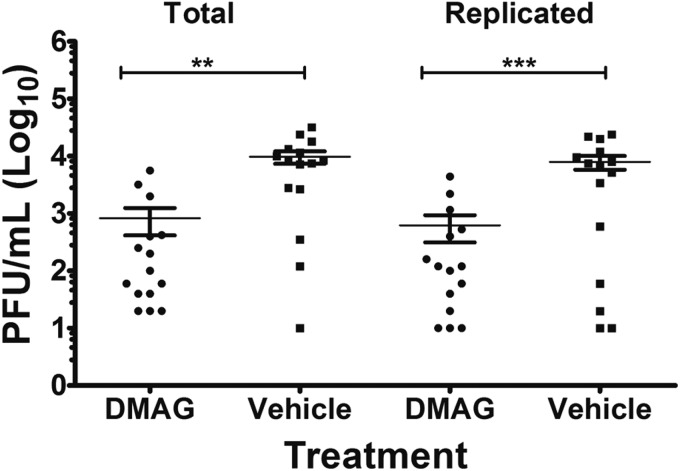
17-DMAG reduces acute MNV-1 infection *in vivo*. MNV-1 replication in mice is inhibited by 17-DMAG treatment. Three groups of five mice each were either treated with 17-DMAG or vehicle in which 17-DMAG was dissolved (0.05% DMSO) and then infected with MNV-1 labeled with neutral red dye. After 24 h, the distal ilea were harvested, and the infectious virus was titrated by a plaque assay either in dark (total) or in light (replicated). Actively replicating, as well as total, MNV-1 titers were significantly reduced. Statistical analysis was performed by using a Student *t* test (ns, nonsignificant; **, *P* < 0.01; ***, *P* < 0.001). Error bars represent the standard deviations.

To determine whether pharmacological inhibition of Hsp90 by 17-DMAG can impact norovirus replication *in vivo*, we used a mouse model of acute MNV-1 infection. Light-sensitive, neutral red-containing virus was used as the inoculum to enable newly replicated virus to be distinguished from input virus (35). Neutral red is a photoactivatable small molecule that can be passively incorporated into virus particles during replication in cell culture. Neutral red-containing virus becomes light sensitive as exposure to light causes cross-linking between the viral RNA genome and the protein coat (42). 17-DMAG was administered to BALB/c mice intraperitoneally as a single bolus prior to infection, and the effect on virus replication in the intestine was examined. The effect of Hsp90 activity inhibition on viral replication in the distal ileum, the primary site of MNV-1 replication *in vivo*, was determined (35, 42). Mice treated with 17-DMAG showed a significant decrease in virus titers compared to vehicle control-treated mice (Fig. 8). These data confirmed a requirement of Hsp90 activity in norovirus replication *in vivo*.

### The HuNoV major capsid protein VP1 is a client protein of Hsp90.

**FIG 9:**
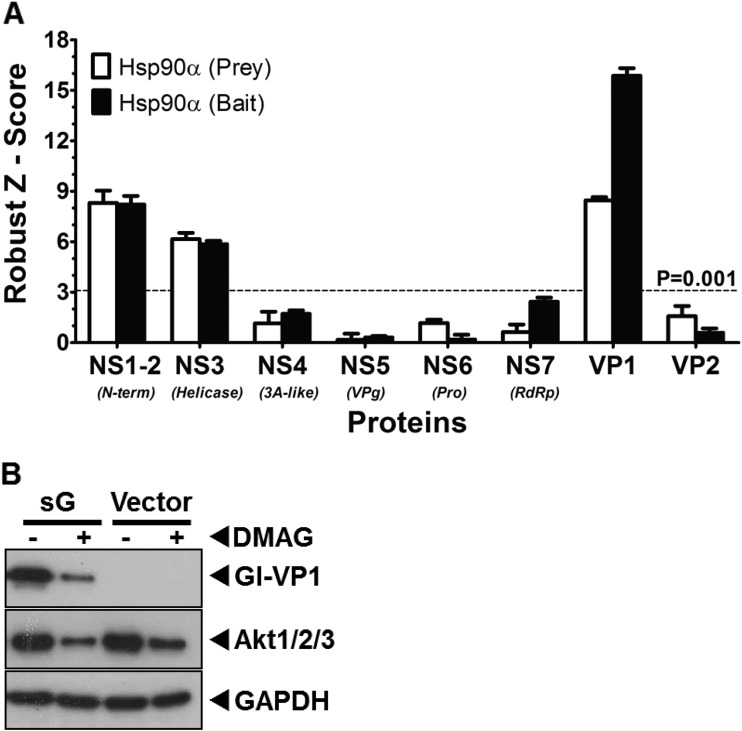
Hsp90 interacts with HuNoV capsid protein, and inhibition of Hsp90 activity leads to its degradation. (A) A Lumier assay was used to detect direct interaction of Hsp90 with HuNoV proteins in 293T cells, as stated in Fig. 6. HuNoV proteins and human hsp90α were expressed in fusion with either protein A (bait) or Renilla luciferase (prey). The expressed proteins were pulled down using IgG, and the interacting fusion Renilla luciferase activity was measured. The robust z scores were calculated to measure the significance of interaction. The assay was performed in both directions. The experiment was performed in triplicate, and the error bars represent the standard deviations. (B) HuNoV structural protein VP1 was expressed in BSR-T7 cells and tested for the ability to degrade in the presence of 17-DMAG. An Hsp90 client protein, Akt, and GAPDH were used as positive and loading controls, respectively.

To determine whether our observations with MNV-1 held true for the HuNoV, we examined whether any of the proteins encoded by the prototype HuNoV, Norwalk virus, also interacted with human Hsp90α using the Lumier assay. The rz scores for each interaction confirmed that interaction of Hsp90α with NS1-2, NS3, and VP1 was statistically significant at a *P* of <0.0001 (data not shown) (Fig. 9A). To confirm that the Norwalk virus VP1 protein was an Hsp90 client protein, we examined the effect of inhibiting Hsp90 activity on VP1 levels when overexpressed in cells. Cells were transfected with a plasmid containing the Norwalk virus subgenomic RNA, encoding both VP1 and VP2, and the effect of 17-DMAG on VP1 levels was examined. As expected, inhibition of Hsp90 activity caused a significant reduction in the levels of the Norwalk virus VP1 protein (Fig. 9B). Taken together, these data independently corroborated that the HuNoV structural protein VP1 is also an Hsp90 client protein.

## DISCUSSION

In our previous report, we had identified Hsp90 as a host factor interacting with the termini of the MNV viral genomic RNA (32). In the present study, we showed that Hsp90 is a potential target for therapeutic intervention against noroviruses. Using Hsp90 inhibitors, we demonstrated that the major role of Hsp90 activity in the norovirus life cycle is during genome encapsidation. We confirmed that the MNV capsid protein VP1 is a client protein of Hsp90 and that inhibition of Hsp90 activity causes VP1 degradation, most likely due to an inability to fold correctly. VP1 degradation results in an inability of MNV-1 genomes to be encapsidated and a significant reduction in the production of infectious virions. We have further shown that HuNoV capsid protein VP1 also interacts with Hsp90 and that inhibition of Hsp90 activity leads to the degradation of HuNoV VP1. Furthermore, we have confirmed Hsp90 activity is required for norovirus replication *in vivo*. Therefore, we propose that Hsp90 may provide a viable therapeutic target for the control of norovirus infections.

Noroviruses are the leading cause of viral gastroenteritis throughout the world (1, 3), and outbreaks are common in semiclosed facilities, including cruise ships, restaurants, schools, hospitals, and caring homes (61, 62). Several factors, including high infectivity of norovirus particles, the persistence of noroviruses in the environment, a lack of lasting immunity, and prolonged shedding of virus from infected individuals, contribute to the explosive nature of norovirus outbreaks (63, 64). HuNoV infection usually presents as an acute and self-limiting disease; however, norovirus infection in infants and young children can develop into more severe gastroenteritis, with symptoms lasting up to 6 weeks (3, 65). Although many vaccine candidates have undergone clinical trials against noroviruses (66–72), the extreme genetic heterogeneity within the virus family, a lack of intergenogroup cross-protection, and rapidly emerging variant strains make generating a cross-protective vaccine a challenging, although not impossible, task. Vaccination is also not a suitable prevention strategy for immunocompromised and immunosuppressed patients, who often suffer from long-term norovirus infections lasting months to years. Therefore, the development of antiviral strategies for such patient cohorts is of great interest. Several efforts have been made to develop antiviral drugs against noroviruses (63, 73–78). Inhibitors targeting the viral genome or proteins have been tested in both cell culture and mouse models for their ability to inhibit norovirus replication (73–77). Small molecule inhibitors of cellular pathways have also been shown to inhibit MNV replication *in vitro* and *in vivo*; hippuristanol, a small molecule inhibitor of eIF4A, has been shown to inhibit MNV RNA translation and virus replication in cell culture (27), whereas a small molecule inhibitor of deubiquitinases can also inhibit MNV replication *in vivo* (78). Despite these efforts, no antiviral therapies are available to treat norovirus infections.

At present, the majority of antiviral strategies target a single viral protein. As a consequence, these antiviral drugs often have a limited range of activity, often targeting only a single virus. The high mutation rates of RNA virus genomes also make many antiviral drugs vulnerable to the rapid emergence of drug resistance. In contrast, strategies designed to target host factors required for viral replication not only have the potential to act on a wider range of viruses due to common mechanisms of replication but also have a higher barrier to the development of resistance. This cellular targeting approach is gaining more enthusiasm and several such host factors, required by many viruses, have now been screened for inhibitors that may have broad-spectrum antiviral activity (27, 78–81).

Our identification Hsp90 using a riboproteomics screen to identify host factors that interact directly or indirectly with the 5′ and 3′ termini of the MNV-1 genome (32) added to the growing list of pathogens that utilize Hsp90 for some aspect of their life cycle. Hsp90 is a highly abundant and evolutionarily conserved molecular chaperone required for the conformational maturation of numerous client proteins, enabling them to carry out their biological functions (33, 82, 83). In addition, Hsp90 is required for the function of a large number of multiprotein complexes (84–86). Hsp90 plays an important role in the replication of a broad range of both DNA and RNA viruses (33). For example, in hepatitis C virus Hsp90 is essential for NS2/3 protease activity and stabilizes the NS3 protein (87, 88), and in human immunodeficiency virus type 1 Hsp90 controls reactivation from latency (89), whereas in poliovirus Hsp90 is required for maturation and processing of the capsid protein precursor (P1) (49). Nonstructural proteins of MNV are encoded in a single open reading frame. However, the levels of some nonstructural proteins were affected more than others (e.g., compare levels of NS1/2 and NS3 in Fig. 5C). However, given a minor effect on viral RNA levels, but a major effect on infectious virus production, we focused our studies on the structural protein VP1. Our data, presented in Fig. 6 and 9, indicate that the structural protein VP1 of MNV and HuNoV are client proteins of Hsp90. The specific sequence requirements for the interaction of Hsp90 with the MNV-1 RNA are beyond the scope of the present study and will require further investigations; however, they fit with previous observations of RNA binding activity observed with bamboo mosaic virus (46). Hsp90 has previously been shown to interact with the genomic RNA of bamboo mosaic virus (46). The interaction of Hsp90 with a structural RNA motif in the 3′UTR of the bamboo mosaic virus genomic RNA conferred its requirement for bamboo mosaic virus but not for bamboo mosaic virus-associated satellite virus. The role of the observed RNA binding activity in the norovirus life cycle also remains to be determined. It is possible that an interaction with viral RNA functions to recruit Hsp90 to newly synthesized viral RNA where it may then recruit VP1 subunits to initiate genome encapsidation. Further studies are clearly required to delineate the precise role of the RNA binding activity.

The requirement of Hsp90 activity in numerous viral life cycles is not surprising, given that viruses typically need to rapidly produce a large quantity of a limited number of structurally complex proteins. For example, a viral structural protein must adopt a precursor conformation that is soluble, but hundreds to thousands of such protein units must then be orderly assembled into a capsid formation in the presence of viral genome that remains infectious despite extreme thermal and chemical conditions (90). The requirement of Hsp90 activity for pathways associated with cancerous cell proliferation has driven the development of inhibitors targeting Hsp90 activity (47, 91). The most effective inhibitors are based on the ansamycin antibiotic GA, which prevents ATP binding to Hsp90, rendering it inactive. There has been enormous interest in the development of Hsp90 inhibitors with an acceptable safety profile for the treatment of several cancers (47), and should any of these possess the correct toxicity profile, we would suggest that there is a possibility to repurpose these Hsp90 inhibitors against noroviruses.

To summarize, we have shown that host chaperone Hsp90 plays an important role in the stability of the norovirus capsid protein and that Hsp90 activity is required for MNV-1 replication *in vitro*, as well as in *in vivo* mouse models. We extended this work to demonstrate that Hsp90 activity is also required for the HuNoV capsid protein stability, identifying Hsp90 as a common cellular component contributing to the norovirus life cycle. Further work is required to explore the utility of Hsp90 as a potential therapeutic target against these clinically important pathogens.
